# Of the Epidemic Catarrh of the Year 1788

**Published:** 1788

**Authors:** George Bew

**Affiliations:** Physician at Manchester


### II. *Of the epidemic Catarrh of the Year* 1788. *By* George Bew, *M. D. Phyſician at Mancheſter.*

THE epidemic catarrh did not make its appearance here, this year, before the latter end of July *. The firſt caſes of it that came under my obſervation were of two gentlemen and a lady, who returned from London the evening of the 28th: they had ſuffered the diſorder ſome time, and were not perfectly recovered.

* Previous to the appearance of this diſeaſe the low nervous fever had prevailed, and in ſome caſes proved fatal. Sore throats of the ulcerous kind had likewiſe affected ſome, particularly children, both in the town and neighbourhood.

On

On the 31ſt of July I was called to viſit another gentleman, who likewiſe had returned from London, in the mail coach, the preceding evening. He had only perceived himſelf attacked the day of his departure; but the fatigue of travelling had very much aggravated his complaints. The day following his brother, a few years older, found himſelf ſeized with the ſymptoms of the diſorder, which continued to the 5th of Auguſt, and were followed by a ſevere fit of the gout, to which he had previouſly been ſubject.

On the 5th of Auguſt a nephew of the above gentleman, a youth about ſixteen years old, was ſuddenly and violently attacked. His ſickneſs was intenſe, and was followed by diſcharges of black and very offenſive ſtools. An antimonial vomit was adminiſtered, which operated effectually, and by the evening of the next day he appeared perfectly recovered.

On the 6th, an older ſiſter, who reſided in the ſame houſe, was ſeverely ſeized with the uſual ſymptoms of the diſeaſe, except the diarrhœa; on the contrary, ſhe had been ſome days coſtive. She continued extremely ill until the 16th, and remained in a debilitated ſtate ſome time after.

 And

And on the 7th another lady, younger ſiſter to the above, was affected in like manner; but an emetic being ſpeedily directed, ſhe recovered in a few days.

I have been particular in diſtinctly noticing the progreſs of the diſeaſe through this family, all of whom were affected, as it ſhews the irregularity which marks not only the commencement, but alſo the duration of this epidemic.

From the 1ſt to the 10th of Auguſt this diſorder proceeded very rapidly; few perſons eſcaped without ſome ſymptoms of the catarrhal fever. All ranks and ages ſeemed alike ſubjected to its influence; nor did the prevalence of any other diſorder whatever repel its attack: on the contrary, I had frequent proofs not only of the continuance even of diſeaſes which originate from particular contagion along with this diſorder, but likewiſe obſerved them to be much aggravated whilſt the ſymptoms of this epidemic remained.

Towards the latter end of Auguſt the diſeaſe became leſs general, and by the beginning of September it ſeemed in a great meaſure to be declined. Some evident ſymptoms of this catarrhal epidemic have, however, at times, continued to appear in a few caſes that have been ſubmitted

ſubmitted to my care; and ſo late as the 15th of October I was called to viſit a gentleman and two of his children in the country, who were all affected with the particular ſymptoms of this diſorder.

The epidemic catarrh of this year generally commenced with a ſenſe of feebleneſs, and an unwillingneſs to any bodily exertion.

A ſtupor prevailed over the ſenſes. In ſome caſes acute pains were felt in the head; but more frequently a dull ſenſation, accompanied with dizzineſs and a painful tightneſs of the breaſt, particularly in reſpiration. A pungent cough, without much diſcharge; frequent ſneezing; painful effuſions from the eyes; together with a fulneſs and ſoreneſs of the mucous membrane of the noſe and throat, and which ſeemed to extend to the ſtomach and along the inteſtinal canal.

Theſe ſymptoms generally became worſe towards evening, and the uneaſy ſenſations more evident. Partial and irregular ſhiverings took place; drowſineſs; wandering pains were felt along the extremities, which were for the moſt part cold and ſhrivelled.

In many caſes the coldneſs of the body and extremities was not ſucceeded by heat, or much evident increafed action of the heart and arteries; but a tingling fenfation was diffufed over the furface of the body, and the exfudatory pores were peculiarly excited. Some degree of delirium fucceeded, which was foon relieved by copious exfudations, and the fweat had often an offenfive fmell. The patient generally had little or no fleep.

The ftimulus to difcharge the urine was frequent, and the urine generally high coloured, in fmall quantities, and of an odour like the fweat, but feldom depofited much fediment. In moft cafes a diarrhœa took place fometimes with the other fymptoms, or foon afterwards. The dejections were frequent, acrid, dark coloured, and very offenfive.

In a few days, if the fweating continued uninterrupted, and the patient fubmitted to proper treatment, the cough, coryza, and excitement of the throat, &c. gradually declined, the urine began to depofit a more perfect fediment, and fleep was reftored, though at firft but flowly and imperfectly: and I generally obferved, that, after the firft return of fleep, the feeblenefs of the fyftem became more evident to the patient, and the fenfes (particularly that of hearing) were much obfcured; moft of the patients that came under my care being affected with deafnefs.

From the commencement to the termination of the difeafe I feldom found the tongue dry or very foul, nor the pulfe increafed much above ten or fifteen ftrokes in a minute beyond its natural quicknefs. Some degree of appetite for food remained with fuch as did not fuffer the diforder feverely, and, unlefs the fweatings were very profufe, the thirft was not immoderate: but the ftrength of the body, and the perfect ftate of the fenfes, (particularly hearing, as has been remarked) was reftored by flow degrees.

The indications of cure, in fubjects where the epidemic catarrh was not accompanied with any other diforder, might readily be diftinguifhed by the fymptoms that have been defcribed; and it muft have been generally obferved, that, in the greater number of cafes, nature alone effected the cure by the ufual increafed evacuations *.

Wherever I could adminifter an antimonial

* This muft have been the cafe with the majority of the lower order of people in this place; for neither the Infirmary reports nor the regifters of mortality were increafed in any comparative degree to the general prevalence of the diforder.

emetic emetic at the first accession of the symptoms, I almost constantly found it put a speedy termination to the disease: first by exonerating the stomach, &c. of the contents, and afterwards either by determining to the surface, so as to assist the sweating, which always accompanied this disorder, or to the intestinal discharges, which, if not immoderate, were favourable to the cure*. I mention antimonial emetics, because I observed the ipecacuanha root not only to operate with greater severity, but to produce some disagreeable effects: and indeed I am inclined to suspect, from attentive observation, that this emetic has been celebrated for efficacy and mildness of operation, which it does not deserve.

After the operation of the emetic, I usually directed neutral draughts to be given in an effervescing state, with a certain quantity of the ætherial spirit of vitriol and tincture of opium; and if the disorder did not retire, I ordered small doses of emetic tartar, with rhubarb, to

* In some cases, as has been remarked, a costiveness took place at the commencement of the disorder, and then it became necessary to administer gentle purgatives; but with the greater number of patients a diarrhœa began with the other symptoms.

be be repeated at intervals, and about a quarter of an hour after each dose to be followed by the neutral draughts. In some cases the opiate was only given with the evening dose. I did not employ blisters, unless the oppression in breathing, the cough, tightness of the breast, and pains in the side or head, were violent, and then I usually gave the camphorated julep along with the neutralised volatile alkali. But I mostly found my patients complain of the disagreeable flavour of the camphor; nor did I observe it produce any extraordinary effects. The blisters I always found serviceable in relieving the pains, the breathing, and the cough.

At first I forbade wine, unless much diluted, or made into whey; but I soon found that a glass or more of wine, according to the age or habits of the patient, might be taken not only with impunity, but with evident advantage: and though I did not allow full meals of animal food, I did not observe that a moderate quantity of broth or boiled meats, where an appetite prevailed, did any injury; on the contrary, I remarked those persons to recover the most speedily who preserved some relish for their food at the usual hours of repast, and especially towards the close of the disorder.

I had,

I had, on former occaſions, remarked the unfavourable conſequences of adminiſtering the Peruvian bark whilſt the excitements of the diſorder continued with any degree of violence; but, on the decline of the diſeaſe, I always found it effectual in aſſiſting the recovery of the patients from the ſtate of debility in which they were, for the moſt part, left after the other ſymptoms had retired; and I likewiſe obſerved it never fail to prevent the return of the diſorder — a circumſtance by no means unuſual with thoſe who did not conclude the cure with ſome preparation of the bark. The mode of giving it I thought moſt eligible, on account of the precarious ſtate of the ſtomach, was in decoction, to which I added a certain quantity either of the acid or ætherial ſpirit of vitriol.

Bleeding was never directed by me, unleſs ſome ſymptoms, beſides thoſe proper to the diſeaſe, abſolutely required it, and then it was employed with the moſt cautious circumſpection. I had frequent opportunities of obſerving the ill conſequences of lowering the ſyſtem by copious bleedings; and though it might afford a tranſient relief of the pains and cough, it always tended to increaſe the debility and anxiety, and prolong the duration of the diſorder.

But

But ſome accidental ſymptoms, particularly in caſes previouſly ſubjected to pulmonary complaints, abſolutely required ſome blood to be immediately taken away. I ſhall offer an inſtance of a gentlewoman of about fifty-five years of age, to whom I was called to viſit in the country, and who had ſuffered much from aſthmatic complaints, for which ſhe had been accuſtomed to loſe blood to relieve her breathing. Some days had elapſed, after ſhe was firſt ſeized, before ſhe apprehended the nature of her diſorder. I found her much diſtreſſed with ſickneſs; ſhooting pains in her head and back, and a pungent fixed one in the left ſide; an inceſſant cough; laborious reſpiration; a quick irregular pulſe; dry ſkin; and a peculiar impatient look denoting the moſt painful ſenſibility. Her breathing was ſo much oppreſſed, that I immediately directed ſome blood to be taken from the arm, which inſtantly relieved her. A large bliſter was applied to the ſide, and medicines of the ſame indications I have before deſcribed were ordered to be given to her. The next day I had the ſatisfaction to find the bliſter had taken proper effect, and that the alarming ſymptoms were moderated. In a few days moſt of them were removed; and by the tenth, from the time I firſt ſaw her, ſhe was perfectly free from every complaint except extreme debility: from this ſhe was in a ſhort time reſtored by means of the Peruvian bark, given as I have before noticed.

Such were the ſymptoms which diſtinguiſhed the epidemic catarrh of this year, and which ſucceeded nearly in the ſame order, though in different degrees of violence, with every one who was ſeized with this diſorder.

But though the diſeaſe prevailed almoſt univerſally, I did not remark that the application for medical aſſiſtance was ſo general as in the year 1782. Perhaps the diſeaſe might not be ſo alarming to the apprehenſion of the Public, on account of its former appearances and effects being ſtill freſh in every one's recollection; together with the moſt ſalutary mode of treating it. Many of the lower order of people conſidered it merely as a cold, and attempted to obviate the debility it induced by drinking and laborious exerciſe. Their attempts were not, however, always ſucceſsful. The powers of the ſyſtem were, for the moſt part, too much affected to allow of much exertion; and thoſe who obſtinately perſiſted to contend with the diſeaſe, diſeaſe, moſt frequently ſuffered for their temerity *.

The conſequ nces of neglect, or improper treatment, particularly of intemperanee and imprudent exertions of ſtrength, did not often paſs with impunity. Fevers of different kinds, but chiefly of the type now diſtinguiſhed by the appellation of *typhus*, were exceedingly prevalent after the epidemic catarrh had, in a great meaſure, ceaſed to be general; but from which, by tracing the ſymptoms, the fevers might uſually be found to have originated. In ſome caſes extraordinary ſymptoms likewiſe followed the imprudent treatment I have juſt mentioned. I ſhall relate an inſtance which came immediately

* I had, of the former, an inſtance in my own family. A ſervant man, about forty years of age, of a ſtrong, healthy conſtitution, whom I uſually employ in the garden, perceiving himſelf ſeized with the diſeaſe along with the other ſervants, determined to try to diſſipate it by hard exerciſe, a mode of cure he had found ſucceſsful in common catarrhal affections, and accordingly ſet himſelf to work on ſome of the moſt laborious parts of his employment: but he ſoon found himſelf too weak to perſevere in his reſolution, and, prudently deſiſting, returned to the houſe, and, with the aſſiſtance of proper treatment, was able to reſume his buſineſs without danger.

under under my notice, and which was very nearly terminating fatally.

A young woman, in the prime of life, and of an excellent conſtitution, who lived in the ſervice of a gentleman's family in the country, was, together with her fellow ſervants, ſeized with the uſual incipient ſymptoms of this diſorder: but, to employ her own language, "ſhe "was reſolved not to yield to it," and reſolutely determined to try whether ſhe could not overcome the diſeaſe by "hard working." She ſet herſelf the moſt laborious employments, ſuch as cleaning the furniture and waſhing the rooms, and continued the conflict the whole day, notwithſtanding the debility and profuſe ſweatings which accompanied her exertions. Late in the evening ſhe changed her cloaths, and waſhed her hands and feet with cold water. The conſequences were ſuch as might be expected. The following account was given me by the gentleman who was firſt called to attend her:—

"Sunday, Auguſt 10th, about noon, found "her affected with an extreme oppreſſion of "the cheſt, and great hoarſeneſs, which might "be heard at ſome diſtance every time ſhe "drew her breath. She complained of an ex- "treme tightneſs of the breaſt, and apprehen-

"ſion

" sion of expanding the chest on account of a

" cough, which was so violent as to create a

" sense of suffocation. Her throat felt dry,

" and she had a pain about the præcordia.

" Her body was tolerably open; but she had

" frequent shiverings, followed by fever."— He took away about ten ounces of blood from her arm, which seemed to give her relief, and then applied to me for my advice and directions.

I saw her the following day. Though her pain had been a little relieved by bleeding, her breathing was become more difficult, and the sense of suffocation and the dryness of her throat were much worse, insomuch, that she said she was afraid either to breathe or speak. Every respiration was accompanied with a peculiar rattling sound, like that of children affected with the *suffocatio stridula*, and was laborious. Her skin felt hot and dry, and was of a very florid red colour; and the pulse small and frequent, about a hundred strokes in a minute. She had sweat a little at different times in the night, which, whilst it continued, relieved her breathing a little; her sickness remained, but she did not swallow with much difficulty. She was attended with the most benevolent care; and and the lady whom ſhe ſerved had very properly kept her body moderately open with clyſters.

During the two following days ſhe continued in much the ſame ſtate, the pulſe ſometimes lowering to 85 or 90. The ſweating was encouraged by diaphoretic medicines; and I directed a large bliſter to be applied to the throat, and another to the region of the ſtomach. She had ſhort ſlumbers, from which ſhe always awoke greatly alarmed and agitated, and always ſhewed an averſion to being removed.

About five in the evening of the fifth day from the attack of the diſorder ſhe complained of unuſual chilneſs of the back and extremities, together with a tremor of the limbs, and ſtill more violent agitation of the whole body than ſhe had ſuffered before. Sudden vivid fluſhings of the face ſucceeded, which were alternated with coldneſs and ſhivering. Her breathing became more laborious, quick and rattling. She had a peculiar quickneſs in her eyes and looks, but was perfectly compoſed and ſenſible, though ſhe uttered the few words ſhe attempted to ſpeak with great difficulty. Her arteries vibrated with too much irregular rapidity to allow me to judge of the pulſe; and ſhe ſeemed reſigned to the termination, which her-

ſelf,

ſelf, as well as her attendants, conſidered as inevitable. The directions I had given had been ſtrictly followed, and her medicines adminiſtered with the greateſt punctuality.

I directed her to be gently raiſed, and, as ſhe now could not ſwallow but with great exertion, I gave her wine by a tea-ſpoonful at a time, allowing her intervals of reſt; at the ſame time I directed her feet and legs to be chafed with warm hands. After about twenty minutes had elapſed, and half a glaſs of wine been gradually ſwallowed, the pulſe beat with leſs irregularity; a warmth began to diffuſe itſelf through the extremities; ſhe was able to ſwallow with leſs difficulty, and ſhe breathed and articulated with more freedom. A copious ſweating followed ſoon after, and the rattling of her breathing ceaſed from that time; her pulſe became tolerably diſtinct and ſoft, and about three in the morning ſhe dropped into a calm ſlumber, which continued many hours.

The next day I found her, in a great meaſure, relieved from her complaints; a gentle diaphoreſis had ſucceeded to her ſlumbers, and ſhe only felt herſelf feeble and diſpoſed to ſleep. A decoction of the Peruvian bark and vitriolic acid were directed to be given her at intervals; and and in a few days ſhe was able to attend to ſuch employments in the houſe as did not require much exertion.

It would be a pleaſing employment to trace any favourable effects reſulting from this almoſt-univerſal epidemic; and in one caſe that came under my notice the diſorder has been followed by flattering circumſtances.

A ſingle lady, of a delicate habit, who, during a long ſeries, had experienced ſevere and frequent attacks of chronic rheumatiſm, and for ſome years paſt had been threatened with hydropic complaints, for which the digitalis had, in vain, been given in every form that could be contrived, but which had been ſuſpended by medicines of gentler operation, was ſeized with the epidemic catarrh the beginning of Auguſt, and the ſymptoms, particularly the ſweating and diarrhœa, were very ſevere. Her evacuations were ſo great, and ſhe was conſequently ſo much enfeebled and exhauſted, that I found it neceſſary to direct cordials along with the other medicines; and as opium in no preparation whatever would agree with her ſtomach, I ordered wine to be often adminiſtered, ſometimes in whey, and ſometimes mixed with water and ſpices.

These

These evacuations continued almoſt inceſſantly from the 7th to the 14th, when they gradually abated, and I had the ſatisfaction to find her leſs debilitated than I expected. In a ſhort time ſhe had recourſe to her former medicines, to which, for a conſiderable time, ſhe had been obliged occaſionally to apply, and ſhe acquaints me, with no ſmall pleaſure, that ſhe finds them prove more efficacious ſince her recovery from the catarrhal diſorder than they had ever been before. Her ſwellings are in a great meaſure ſubſided; her appetite and ſtrength much reſtored; and ſhe is freed from a painful depreſſion and anxiety that had, during a long time, conſtantly embittered her life.

I have hitherto taken no notice of the weather previous to and during the prevalence of this epidemical diſeaſe in Mancheſter: nor did I remark any particular ſtate in the ſenſible qualities of the atmoſphere that alone could be ſuppoſed to produce or even propagate it. Perhaps catarrhal diſeaſes, in general, are not ſo much derived from a certain irregularity and moiſture of the air as has been formerly imagined; the different recurrences of this diſeaſe having happened in no certain ſtate or quality of the air, nor particular time of the year*: and I am led to believe that the epidemic catarrhs of former periods muſt have been generated and diffuſed by other means than the influences of ſeaſons and atmoſphere. In the deſcription of the weather prefixed by Dr. Whytt to the accounts of the epidemic which prevailed in the ſouthern parts of Scotland during the year 1758†, and which ſeems to have reſembled the epidemic catarrhs of 1775, 1782, and 1788, the ſtates of the atmoſphere do not appear to have had any thing more uncommon than might be expected from the ſituation of a country ſurrounded by the ocean, interſected with rivers, lakes, and large arms of the ſea, with numerous ports and harbours where ſhips arrive from all countries. That it did not extend much beyond the confines of Scotland might poſſibly be owing to the intercourſe between

* In England,

The epidemic catarrh of 1775 began in November;

Weather warm and moiſt.

——————— of 1782 began in May;

Weather cold and wet.

——————— of 1788 began in June and July;

Weather not uncommon.

† Vide Medical Obſervations and Inquiries, Vol. II.

the the formerly-divided nations not being, by any means, ſo general as it is at preſent.

But not only in the above-mentioned, but in all the epidemical catarrhs, of which we have any clear and rational accounts, there ſeems to have occurred a ſtriking reſemblance in the principal ſpecific ſymptoms, as well as in the commencement, progreſs, and decline of the ſeveral periods of the diſeaſe *.

The memorable *Sudor Anglicus* was probably only a more malignant ſpecies of the ſame epidemic diſorder; the chief ſymptoms, according to the beſt accounts tranſmitted to us, bearing a ſtrong reſemblance to the catarrhal epidemic fevers of our own times. Like theſe, it was almoſt univerſally contagious †, was attended with

* The plague of Athens, according to the celebrated account of Thucidides, began with ſneezing, hoarſeneſs, cough, pains in the breaſt, and vomiting, &c.

† "Tanta fuit febris hujus malignæ initio truculentia, "ut quamprimum urbem aliquam invaderet, ſingulis diebus "quingentos aut ſexcentos occuparet. . . . . Correpti, ſtatim languore diſſolvebantur & animo deficiebant cum ſummo virium languore, inquietudine καρδιωγμῷ, capitis dolore, "pulſu crebro, celeri, inæquali, cordiſque palpitatione maximâ. . . . Sudore difluebant perpetuo & copioſo."—Sennert. T. II. p. 207.

"Morbus with great languor and proſtration of ſtrength, and was of tranſient duration. It is ſaid " its " manner of attack was always the ſame; that " in its different recurrences the ſymptoms were " the ſame; and that it rarely ſtaid more than " a week in a place *." Even its malignity might be aggravated by the deplorable ſtate of the nation, the diſtraction of the times, and the barbarous treatment of the ſick. Fatal as the diſeaſe itſelf was, we are told that " more were " obſerved to die by the hands of empirics " than by the diſorder †."—Slaves to credulity and the prejudice of opinion, the phyſicians, or more properly the pretenders to phyſic, of thoſe times, ſeem to have ſtudied to counteract and ſubdue, rather than follow and aſſiſt, the ſalutary efforts of nature. The ſweating ſickneſs had made repeated viſits, and " killed more " than the nation was ſuppoſed to contain at " one time ‡," before they perceived and avail-

" Morbus iſte brevis ſortitus & periodus, tam in morbi ipſius criſi, tam in tempore duratione ipſius."—Lord Bacon, Vol. V. p. 231.

* Vide Hiſtory of Air, Epidemics, &c. Vol. I. p. 209.

† Ibid.

‡ Ibid.

ed themſelves of the means the diſeaſe itſelf indicated as the proper mode of cure, and which they too often defeated by fantaſtical forms, and rendered fatal by purſuing with abſurd and mercileſs rigour *.

Thoſe authors who have written on the hiſtory and cure of the Sudor Anglicus have uniformly attributed the cauſe of it to ſome pecu-

* " ——— primum vero ex Britannicis locis terras maritimas Hollandiæ ac Zelandiæ pervagata, Antwerpiam venit, ac celerrime in Flandriam, totamque Brabantiam ſparſa, uno die ſudoris infanda eluvie hominum multa millia ſuffocabat. His aderat ea dementia, ut ſeſe lactis ac linteis inſui paterentur, omni arte ac vi eliciendum ſudorem arbitrati: .... Imo interea dum alter alterum ſtrangularet, qui præſentes aderant, mutuo hortabantur ne ſe negligerent, urgente morbi ferociâ, ne victi pretio, aut precibus ſe ante tempus liberarent. Verum quando ad illos ordo pervenerat, ut ſudandi tempus videretur, conſuti ſimiliter, & violenter operti clamitabant miſeré, obteſtabantur Deum atque hominum fidem, ſeſe dimitterent, ſe ſuffocari injectis molibus, ſeſe vitam in ſummis anguſtiis exhalare; ſed aſſiſtentes, has quærelas ex rabie proficiſci, medicorum opinione perſuaſi, urgebant continué, uſque ad 24 horas, id enim erat ſudandi ſpatium præfinitum, ante cujus circuitum evaſere quam pauciſſimi; tertia mortalium pars errore proprio, atque inſaniâ miſere ſtrangulata, vitaque cum gemitu fugit indignata ſub umbras." — *Gem. coſm. (de Sudore Angl. ap. Schenk.)* lib. i. cap. 8.

liar liar ftates of the feafons and atmofphere *, and feem to have bufied themfelves in contriving curious proceffes to alter and amend the air, inftead of attending to the actual operations and progrefs of the difeafe †. The progrefs of the contagion may, perhaps, be more certainly traced by attending to the hiftorical tranfactions of the times. The fweating ficknefs is faid to have firft appeared in the army of the Earl of Richmond, afterwards Henry the Seventh, who landed at Milford on the 7th of Auguft, 1485, with a few French foldiers. They are defcribed as being ill armed; and in all probability were no better furnifhed with raiment, food, or other accommodations. Little attention could be paid either to cleanlinefs or health during the fhort and perilous progrefs of the army through Wales to the memorable fields of Bofworth.

* "In Britannia infula genus eft peftilentis febris, & con- "tagiofæ....eft autem non contagiofa folum de uno in "alium, fed vagatur etiam de civitate in civitatem. Qua- "propter in vitio aëris præcipue enafci cenfendum eft."—— *Vide* Fracaft. *de Morb. contag.* Lib. ii. c. 5.

† "Quapropter venenofam iftam aëris qualitatem, fi lubet "aut prohibere (id quod multo eft optabilius) aut profli- "gare," &c.— Fernelius, *Confil.* 69. *ad peft. Angl. precaut. emend. aeris.*

The

The decisive battle was fought on the 22d of the same month. Henry and his followers arrived in London on the 29th, and on the 22d of the next month the sweating sickness was epidemical in that city.

But though the epidemical catarrhs, in whatever manner or from whence they may originate, evidently seem to be contagious from infection derived from the human body, rather than from any peculiar state of the atmosphere; yet the various states of the atmosphere may have great influence in promoting or retarding their progress and malignity. I observed those patients recover the soonest who were confined to warm, but not very close rooms; and, on the contrary, exposure, especially to the nocturnal air, always aggravated the symptoms of the disorder. Of this I had myself experience. Though I daily attended a considerable number of the sick, I escaped without contracting the disorder until the 14th of August. In the evening of that day, finding myself affected with lassitude, coryza, &c., I took an antimonial emetic, and was about to follow it with some diaphoretic medicine, and retire to bed, when I was summoned to visit a person in the country with such earnestness, that I thought it my duty to comply.

ply. I had about five miles to proceed, and my attendance was neceſſary ſome hours; and though I took every precaution to ſecure myſelf from the influence of the air, which was moiſt and warm, I had convincing proofs of the injury it occaſioned. I had the ſymptoms common to this diſorder with greater ſeverity than I obſerved in any other caſe: but they afforded me the means of marking the nature and progreſs of the diſeaſe with a degree of minuteneſs that I might not perhaps have ſo fully derived from the moſt attentive obſervation of others.

*Mancheſter,*

Nov. 29, 1788.

